# Radiation Protection in the Orthopedics Department: Insights From a Cross-Sectional Study

**DOI:** 10.7759/cureus.75940

**Published:** 2024-12-18

**Authors:** Ahmad Abanomy

**Affiliations:** 1 Radiological Science Department, College of Applied Medical Science, King Saud University, Riyadh, SAU

**Keywords:** occupational exposure, orthopedics, orthopedic surgeons, personal dosimeter, radiation protection

## Abstract

Objective: This study assesses radiation protection practices and knowledge among orthopedic surgeons in Saudi Arabia.

Methods: This cross-sectional study surveyed orthopedic surgeons in Saudi Arabia using an online standardized and pre-tested questionnaire. The data were analyzed using statistical software.

Results: The cross-sectional study was conducted among 102 participants. This survey revealed significant gaps in understanding safety protocols for radiation, as formal training in radiation protection was provided to only 3.9% of surgeons, whereas 7.8% had a basic awareness of the ALARA (as low as reasonably achievable) principle. Even though 67.6% of respondents reported the regular use of lead aprons, other radiation protective equipment, such as leaded eyeglasses and thyroid shields, was less frequently or rarely used; moreover, 80.4% of participants did not use dosimeters to monitor their radiation dose.

Conclusion: The findings of the study comply with the global trends that may be traced in the investigations of other studies where inconsistent use of protective equipment and a lack of corresponding knowledge were prevalent. The current investigation accentuates the importance of mandatory radiation safety training and regular monitoring of the enforcement of protective equipment use; moreover, prioritizing these measures can protect surgeons from the enduring risks of radiation exposure, such as cataracts, cancer, and other radiation-induced disorders.

## Introduction

Exposure to ionizing radiation is essential in several surgical professions that use radiologic imaging technology, including fluoroscopy, intraoperative CT for localization, radiopaque dye for vascular visualization, and confirmation of orthopedic alignment or device placement. Orthopedic procedures integrate technology with medical expertise, allowing surgeons to diagnose or treat various conditions [1]. The radiation exposure for orthopedic surgeons has consistently risen since the introduction of fluoroscopy in the 1950s, especially during minimally invasive operations like femoral nail placements. Nonetheless, the extensive application of X-rays in orthopedic procedures poses several health risks [2].

The health effects of radiation exposure can be categorized as stochastic or deterministic. The deterministic effect occurs after exceeding a threshold dose, and the extent of the effect is directly proportional to the radiation dose. Typical examples include radiation-induced skin erythemas and cataracts. The stochastic effect occurs randomly, with its probability increasing with the dose, but its severity remains dose-independent. This includes radiation-induced primary and secondary cancers, as well as heritable mutations [3].

Radiation protection practices in orthopedic surgeries include the application of the ALARA (as low as reasonably achievable) principle, which includes a decrease in radiation time, an increase in the distance away from a radiation source, and the application of dose limitation. Other practices include the use of personal protective equipment (PPE) while operating ionizing radiation equipment [4].

The objective of this study is to examine the radiation protection knowledge and practices of orthopedic surgeons in the operating room. The research findings could significantly influence policy and regulation by allowing national health authorities to revise and update guidelines, therefore improving radiation safety protocols in orthopedic surgery departments.

## Materials and methods

Study design and setting

A cross-sectional observational study was conducted from January to May 2024 to evaluate the radiation protection practices of orthopedic surgeons currently practicing in Saudi Arabia. The study was approved by the Institutional Review Board at King Saud University (E-24-8468; date of approval: January 23, 2024).

Study population

A standardized and pre-tested self-administered questionnaire was produced using online Microsoft Forms (Microsoft Corporation, Redmond, Washington) and distributed via various social media platforms (WhatsApp, Twitter, etc.). As of 2022, there were 3489 orthopedic surgeons in Saudi Arabia [5]. To determine the sample size for a 95% confidence level and a 0.05 margin of error, given the total population size of 3,489, the sample size was approximately 347. The inclusion criteria were orthopedic surgeons in Saudi Arabia; orthopedic surgeons who were not practicing in Saudi Arabia were excluded from the study.

Study questionnaire

The research objective, study aim, and consent form were all specified in a clear and concise manner at the beginning of the questionnaire. This was implemented to ensure that the participants understood the purpose of the research and could participate voluntarily. In order to protect the participants' privacy and safeguard their confidentiality, this study did not gather their names. In general, the survey was divided into three sections. The first section included a series of questions concerning the participants' demographic information. The second and third sections included questions about their understanding and expertise related to orthopedic procedures and the radiation protection associated with operations.

Four experts from various fields reviewed and approved the questionnaire to ensure that the research objective was satisfied. The research purpose and online questionnaire link were emailed to each expert. Upon reviewing the questionnaire, they answered the questions. Subsequently, the same four individuals met to discuss their opinions and establish a solution. Consequently, 10 participants from the defined population evaluated the questionnaire's language clarity in a pilot survey. The study version of the questionnaire was developed using pilot survey feedback. This version featured clear, basic, and comprehensible questions designed to achieve the study's aim.

Statistical analysis

Responses were recorded onto a dedicated Microsoft Excel sheet and exported to IBM SPSS Statistics for Windows, Version 27 (Released 2020; IBM Corp., Armonk, New York) [6]. The variables were presented as frequencies with percentages, and the chi-square test was used to uncover any statistically significant differences between participants who consistently used PPE and those who did not always use PPE based on gender, age group, years of practice, position, and familiarity with the ALARA principle. A p-value of less than 0.05 was considered statistically significant for all analyses.

## Results

A total of 102 respondents participated in the study, the vast majority of whom were male (n = 94). Most of the participants were aged between 25 and 35 years old (58%), and the majority had 5-10 years of experience (36%). At the time of the survey, the most common positions were consultant and resident doctor (n = 40). Only four respondents had formal training in radiation protection, while eight were familiar with the ALARA principle. Table 1 shows the demographic and professional characteristics of the participants.

**Table 1:** Demographic and professional characteristics of the participants ALARA: as low as reasonably achievable

Variable		Frequency (%)
Gender	Male	94 (92.2)
	Female	8 (7.8)
Age group	25–35	59 (57.8)
	36–45	33 (32.4)
	46–55	7 (6.9)
	> 55	3 (2.9)
Years of practice (including residency)	< 5	23 (22.5)
	5–10	37 (36.3)
	11–15	22 (21.6)
	16–20	14 (13.7)
	21–25	2 (2.0)
	> 25	4 (3.9)
Current job title	Consultant	40 (39.2)
	Assistant/Associate Consultant	15 (14.7)
	Fellow doctor	6 (5.9)
	Specialist doctor	5 (4.9)
	Resident doctor	40 (39.2)
Sector	Government	64 (62.7)
	Private	1 (1.0)
	Both	37 (36.3)
Formal training in radiation protection	Yes	4 (3.9)
	No	98 (96.1)
Familiarity with the ALARA principle	Yes	8 (7.8)
	No	94 (92.2)
Days per week of surgery requiring intraoperative imaging	1	14 (13.7)
	2	31 (30.4)
	3	37 (36.3)
	4	13 (12.7)
	5	0
	More than 5	7 (6.9)
Number of surgeries performed in the operating room	1–3	69 (67.6)
	4–6	25 (24.5)
	7–8	6 (5.9)
	9–11	0
	More than 11	2 (2.0)
Hours per surgery/procedure	Less than 1	11 (10.8)
	1–2	35 (34.3)
	3–4	33 (32.4)
	5–6	11 (10.8)
	More than 6	12 (11.8)
Percentage of procedures requiring X-ray equipment	All procedures	40 (39.2)
	More than 50%	53 (52.0)
	25–50%	6 (5.9)
	Less than 25%	3 (2.9)

**Figure 1:**
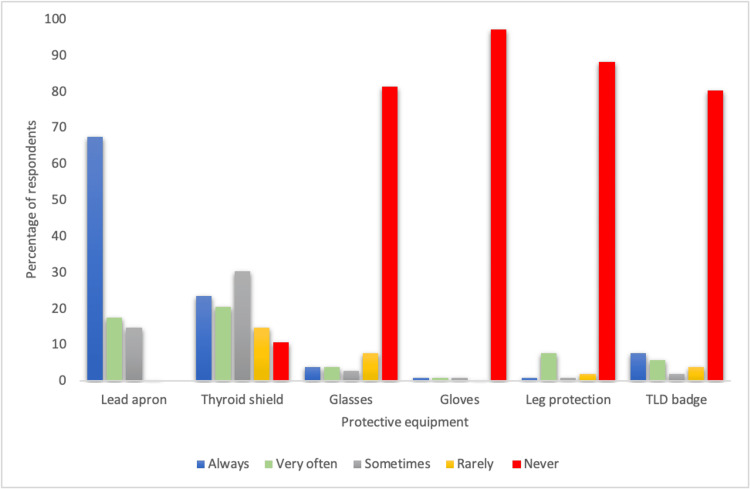
Bar chart illustrating the use of radiation protective equipment and TLD badges among the participants TLD: thermoluminescent dosimeter

Regarding the use of protective equipment, most respondents reported always using a lead apron (68%), while only 24% always used a thyroid shield. Furthermore, a vast majority reported never using radiation protection glasses (81%), gloves (97%), protection for legs (88%), and TLD (thermoluminescent dosimeter) badges (80%) (Table 2) (Figure 1). The comparison of radiation PPE usage based on gender did not reveal any statistically significant differences between those who always used PPE and those who did not always use it (Table 3).

**Table 2:** Breakdown of radiation protective equipment and TLD badge use among participants

Question	Response	Frequency (n)	Percentage (%)
During procedures, do you use a lead apron?	Always	69	67.6
	Very often	18	17.6
	Sometimes	15	14.7
	Rarely	0	0
	Never	0	0
During procedures, do you use a thyroid shield?	Always	24	23.5
	Very often	21	20.6
	Sometimes	31	30.4
	Rarely	15	14.7
	Never	11	10.8
During procedures, do you use radiation protection glasses?	Always	4	3.9
	Very often	4	3.9
	Sometimes	3	2.9
	Rarely	8	7.8
	Never	83	81.4
During procedures, do you use radiation protection gloves?	Always	1	1.0
	Very often	1	1.0
	Sometimes	1	1.0
	Rarely	0	0
	Never	99	97.1
During procedures, do you use radiation protection for your legs?	Always	1	1.0
	Very often	8	7.8
	Sometimes	1	1.0
	Rarely	2	2.0
	Never	90	88.2
During procedures, do you use the thermoluminescent dosimeter (TLD) badge (or any type of dosimeter)?	Always	8	7.8
	Very often	6	5.9
	Sometimes	2	2.0
	Rarely	4	3.9
	Never	82	80.4

**Table 3:** Comparison of the use of radiation protection equipment and TLD badge (always use vs. not always use) based on gender

Question	Males	Females	Chi-Square	p-value
During procedures, do you use a lead apron?	66 (70.2)	3 (37.5)	3.60	.058
During procedures, do you use a thyroid shield?	21 (22.3)	3 (37.5)	.94	.332
During procedures, do you use radiation protection glasses?	4 (4.3)	0	.35	.552
During procedures, do you use radiation protection gloves?	1 (1.1)	0	.09	.769
During procedures, do you use radiation protection for your legs?	1 (1.1)	0	.09	.769
During procedures, do you use the thermoluminescent dosimeter (TLD) badge (or any type of dosimeter)?	7 (7.4)	1 (12.5)	.26	.610

In terms of age groups, the use of lead apron (p = .011), thyroid shield (p < .001), and TLD badge (p < .001) was more common among older age groups (46-55 and >55 years) compared to the younger age groups. Similarly, the use of radiation protective glasses was most common in those aged >55 years (p < .001) (Table 4).

**Table 4:** Comparison of the use of radiation protection equipment and TLD badge (always use vs. not always use) based on age group

Question	25–35	36–45	46–55	>55	Chi-Square	p-value
During procedures, do you use a lead apron?	43 (72.9)	16 (48.5)	7 (100)	3 (100)	11.06	.011*
During procedures, do you use a thyroid shield?	7 (11.9)	9 (27.3)	5 (71.4)	3 (100)	23.39	< .001*
During procedures, do you use radiation protection glasses?	0	2 (6.1)	0	2 (66.7)	34.44	< .001*
During procedures, do you use radiation protection gloves?	0	1 (3.0)	0	0	2.11	.550
During procedures, do you use radiation protection for your legs?	0	1 (3.0)	0	0	2.11	.550
During procedures, do you use the thermoluminescent dosimeter (TLD) badge (or any type of dosimeter)?	0	3 (9.1)	3 (42.9)	2 (66.7)	31.33	< .001*​​​​​​​

In terms of years of practice, the use of a thyroid shield was reported to be more common in more experienced participants, with those having >25 years of practice having the highest reported use (100%) (p = .001). A similar trend was observed for the use of TLD badges, with practitioners having 21-25 years and over 25 years of experience reporting the highest usage (50%) (p = 0.002) (Table 5). Based on job title, fellows (68%) and consultants (43%) reported to have the highest use of a thyroid shield (p < .001) (Table 6). Finally, based on familiarity with the ALARA principle, all those familiar with the ALARA principle reported always using lead aprons, compared to only 65% of those who were not familiar with the principle (p = .042) (Table 7).

**Table 5:** Comparison of the use of radiation protection equipment and TLD badge (always use vs. not always use) based on years of practice

Question	<5	5–10	11–15	16–20	21–25	>25	Chi-Square	p-value
During procedures, do you use a lead apron?	18 (78.3)	27 (73.0)	10 (45.5)	8 (57.1)	2 (100)	4 (100)	10.19	.070
During procedures, do you use a thyroid shield?	1 (4.3)	8 (21.6)	5 (22.7)	5 (35.7)	1 (50.0)	4 (100)	19.72	.001*
During procedures, do you use radiation protection glasses?	0	1 (2.7)	0	2 (50.0)	0	1 (25.0)	10.77	.056
During procedures, do you use radiation protection gloves?	0	1 (2.7)	0	0	0	0	1.77	.879
During procedures, do you use radiation protection for your legs?	0	1 (2.7)	0	0	0	0	1.77	.879
During procedures, do you use the thermoluminescent dosimeter (TLD) badge (or any type of dosimeter)?	0	1 (2.7)	2 (9.1)	2 (14.3)	1 (50.0)	2 (50.0)	18.91	.002*

**Table 6:** Comparison of the use of radiation protection equipment and TLD badge (always use vs. not always use) based on position/job title

Question	Resident	Fellow	Assistant/Associate Consultant	Consultant	Specialist	Chi-Square	p-value
During procedures, do you use a lead apron?	26 (72.2)	6 (100)	10 (66.7)	23 (57.5)	4 (80.0)	5.45	.244
During procedures, do you use a thyroid shield?	1 (2.8)	4 (66.7)	2 (13.3)	17 (42.5)	0	25.23	< .001*
During procedures, do you use radiation protection glasses?	0	0	1 (6.7)	3 (7.5)	0	3.58	.466
During procedures, do you use radiation protection gloves?	0	0	0	1 (2.5)	0	1.57	.815
During procedures, do you use radiation protection for your legs?	0	0	0	1 (2.5)	0	1.57	.815
During procedures, do you use the thermoluminescent dosimeter (TLD) badge (or any type of dosimeter)?	0	0	3 (20.0)	5 (12.5)	0	8.27	.082

**Table 7:** Comparison of the use of radiation protection equipment and TLD badge (always use vs. not always use) based on familiarity with the ALARA principle

Question	Familiar With ALARA	Not Familiar With ALARA	Chi-Square	p-value
During procedures, do you use a lead apron?	8 (100)	61 (64.9)	4.15	.042*
During procedures, do you use a thyroid shield?	4 (50.0)	20 (21.3)	3.38	.066
During procedures, do you use radiation protection glasses?	1 (12.5)	3 (3.2)	1.70	.193
During procedures, do you use radiation protection gloves?	0	1 (1.1)	.09	.769
During procedures, do you use radiation protection for your legs?	0	1 (1.1)	.09	.769
During procedures, do you use the thermoluminescent dosimeter (TLD) badge (or any type of dosimeter)?	2 (25.0)	6 (6.4)	3.54	.060

## Discussion

In the current study, sufficient gaps in radiation protection familiarity and practices among Saudi Arabian orthopedic surgeons were revealed; these findings are consistent with similar studies conducted in countries such as South Korea and India [7,8]. In this context, particular emphasis should be put on the fact that even though the use of radiologic imaging modalities, such as fluoroscopy, has significantly increased in the area of orthopedic surgery, the general understanding and practical implementation of proper measures of radiation safety is still limited [7-9].

The majority (n = 82, 80%) of orthopedic surgeons in this study used intraoperative imaging between one and three days per week. A total of 94 surgeons reported that they performed, on average, one to six surgeries that required intraoperative imaging, with more than half (n = 68, 66%) having an average of four hours or less per surgery. This is consistent with the findings of a previous study conducted on Brazilian orthopedic surgeons [10].

Female orthopedic surgeons are four times more likely to develop cancer than other doctors [11]. The correlation between radiation exposure and cancer risk is well established; however, the consequences of extended exposure to low-level radiation remain uncertain. Three established methods for reducing the effects of radiation include using protective garments and equipment, increasing the distance from the radiation source, and minimizing exposure duration [11-14].

One of the most critical issues stressed in the survey was the lack of formal training on radiation protection since formal radiation safety education was provided to only 3.9% of respondents, and only 7.8% had knowledge of the ALARA principle; these findings have similarities with the results from India, where 82% of orthopedic surgeons did not have knowledge on the recommended limits on annual radiation exposure [8]. In the same manner, it was indicated in a South Korean study that a substantial percentage of orthopedic surgeons were not connected to national systems of radiation monitoring, designating a global tendency of limited awareness and knowledge regarding radiation-related hazards [7].

Furthermore, it is essential to note that the gaps in awareness have extended beyond elementary knowledge, as may be traced from the fact that many surgeons in both India and Saudi Arabia were unaware of the concepts of critical safety, such as proper positioning of the C-arm and the suitable distance that should be maintained from the sources of radiation [8,15]. In an Indian study, 44% of respondents had no awareness of the safe distance essential for minimizing radiation exposure, and 30% were not aware of the principles of C-arm positioning [8]. The importance of structured and mandatory education on radiation safety is accentuated by these gaps, particularly in relation to principles of minimizing exposure and the fluoroscopic equipment's safe use. In Canada, a study revealed that orthopedic surgeons were uneducated about the potential risk of cataracts from radiation exposure, with 75% not knowing the radiation dose limits [16]. Another study conducted in Turkey revealed insufficient understanding among orthopedic surgeons of the applications and hazards associated with fluoroscopy and radiation protection [17].

The risks posed by inadequate knowledge are furthermore exacerbated by the inconsistent use of protective equipment; in the current study, it was revealed that 67.6% of respondents constantly use lead aprons, whereas other important protective equipment, such as thyroid shields and leaded eyeglasses, were less frequently or rarely used. Consistently, in India, thyroid shields were never used by 83% of orthopedic surgeons, and none of them used leaded eyeglasses [8]. In contrast, in South Korea, lead aprons are consistently worn by only 52% of the respondents, and thyroid shields are used by 29% [7].

The study clearly indicates the interrelation between adherence to radiation safety practices and experience. Remarkably, protective equipment, such as dosimeters and thyroid shields, is more likely to be used by more experienced and older surgeons. Similar trends were observed in South Korea, where senior surgeons followed radiation safety protocols more frequently than their younger colleagues and residents [7]. Therefore, the need for early structured training is accentuated by the higher use of protective gear by experienced surgeons in the current study.

While lead aprons are used during C-arm-assisted orthopedic surgeries, they do not offer sufficient protection for the thyroid. A thyroid shield could reduce the total radiation exposure by approximately 50%. The persistent disregard for thyroid shields is concerning because long-term radiation exposure may cause thyroid cancer [18]. This tendency reflects a prevalent lack of compliance with protective measures; moreover, the discomfort and irritating heat caused by the use of protective equipment, such as thyroid shields, was indicated as one of the reasons for noncompliance, furthermore causing its improper use, leaving surgeons vulnerable to avoidable radiation [7,19].

The alarming issue identified within the current study is the low use of dosimeters (TLD badges); remarkably, it was reported by 80.4% of the study respondents that they have never used the dosimeter. Similar tendencies may be seen in the studies pertaining to India (11% regular usage) and South Korea, where less than 25% of surgeons used a dosimeter [7,8]. Consequently, it is possible to claim that the risk of surpassing recommended exposure limits without detection is heightened by the lack of monitoring. The annual limit for occupational radiation exposure is set at 20 millisieverts (mSv) per year in the Saudi Nuclear and Radiological Regularity Commission (NRRC) guidelines [20], whereas obedience to this limit remains low on the global scale. This tendency, in turn, accentuates the need for firmer enforcement of mandatory dosimeters and the use of protocols for radiation safety in orthopedic surgery.

The challenges pertaining to radiation safety detected in the scope of this study are not exceptional to Saudi Arabia since the analogous issues were indicated by the scholars in South Korea and India; remarkably, only 56% of surgeons in India were conscious of the safe distance from the sources of radiation, and 60% understood the interrelation between radiation exposure and magnification [7,8]. Despite the prevalent use of fluoroscopy technology in South Korea, the compliance of such procedures with the contemporary protocols on radiation safety is still inconsistent [7]. Therefore, it is indicated by these global trends that orthopedic surgeons face common difficulties while adopting effective practices on radiation protection, accentuating the need for stricter enforcement and complex radiation safety guidelines aimed at minimizing radiation risks to orthopedic surgeons.

Limitations and future research

This cross-sectional research has several limitations. First, it focused on surgery time, which may not adequately represent radiation exposure; acquisition time would accurately reflect radiation exposure time. Second, the sample size of 102 surgeons may restrict generalizability, and a larger sample size might effectively represent Saudi Arabian radiation protective protocols. However, this study provided an initial perception of orthopedic surgeons' practices and training concerns and suggested further research initiatives. Another limitation of this study is its reliance on self-reported data, which may have introduced response bias, since participants may overestimate or underestimate their adherence to radiation protective practices. The research aimed for nationwide representation; nevertheless, the lack of exact regional data constrained the comprehension of any local variations in practices. Future research may further discuss this problem by collecting region-specific data.

## Conclusions

The findings of this study indicate the urgent need for enhanced training on radiation protection and corresponding practices among Saudi Arabian orthopedic surgeons. The global trends traced in the studies from South Korea and India reflect these knowledge gaps and inconsistent use of protective equipment and dosimeters. In the current study, the majority of orthopedic surgeons reported never wearing personal radiation dosimeters, indicating a deficiency in monitoring and understanding of personal radiation exposure levels that may lead to increased occupational radiation exposure. Enforcing the use of personal dosimeters might foster a safety culture that guarantees that surgeons are aware of their radiation exposure, hence reducing possible long-term health hazards.

Hence, in order to address these risks, there is a need to prioritize mandatory education on radiation safety and enforce compliance with protective measures by the healthcare authorities. Furthermore, adequate protective equipment should be provided by the hospitals, as well as the management of the healthcare facilities should ensure regular monitoring of radiation exposure through the use of dosimeters and, eventually, develop and implement into the practice structured programs on radiation safety training, predominantly for residents and younger surgeons.
